# Early Screening for Gestational Diabetes Using IADPSG Criteria May Be a Useful Predictor for Congenital Anomalies: Preliminary Data from a High-Risk Population

**DOI:** 10.3390/jcm9113553

**Published:** 2020-11-04

**Authors:** Agnieszka Zawiejska, Katarzyna Wróblewska-Seniuk, Paweł Gutaj, Urszula Mantaj, Anna Gomulska, Joanna Kippen, Ewa Wender-Ozegowska

**Affiliations:** 1 Department of Reproduction, Poznan University of Medical Sciences, 60-512 Poznan, Poland; pgutaj@o2.pl (P.G.); urszula.mantaj@gmail.com (U.M.); eozegow@ump.edu.pl (E.W.-O.); 2 Department of Newborns’ Infectious Diseases, Poznan University of Medical Sciences, 60-512 Poznan, Poland; kwroblewska@post.pl; 3 Student Scientific Society—Student Research Group, Department of Reproduction, Poznan University of Medical Sciences, 60-512 Poznan, Poland; anagomulska@gmail.com (A.G.); joannakippen@gmail.com (J.K.)

**Keywords:** hyperglycemia in pregnancy, early gestational diabetes, fetal malformations

## Abstract

Background: Our aim was to investigate whether the International Association of the Diabetes and Pregnancy Study Groups (IADPSG) glycemic thresholds used for detecting hyperglycemia in pregnancy can be predictive for malformations in women with hyperglycemia detected in early pregnancy. Methods: a single-center, retrospective observational trial of 125 mother-infant pairs from singleton pregnancies with hyperglycemia according to the IADPSG criteria diagnosed at the gestational age below 16 weeks. Glucose values obtained from 75-g OGTT (oral glucose tolerance test) were investigated as predictors for congenital malformations in newborns. Results: Characteristics of the cohort: maternal age: 31.5 ± 5.2, pre-pregnancy body mass index (BMI) ≥ 30 kg/m^2^: 42.0%, gestational age at diagnosis (weeks): 12.0 ± 4.0, and newborns with congenital malformations: 8.8%. Fasting blood glycemia (FBG) and HbA1c (Haemoglobin A1c) at baseline significantly predicted the outcome (expB: 1.06 (1.02–1.1), *p* = 0.007 and expB: 2.05 (1.24–3.38), *p* = 0.005, respectively). Both the fasting blood glucose (FBG) value of 5.1 mmol/dL (diagnostic for gestational diabetes mellitus (GDM)) and 5.5 mmol/dL (upper limit for normoglycemia in the general population) significantly increased the likelihood ratio (LR) for fetal malformations: 1.3 (1.1; 1.4) and 1.5 (1.0; 2.4), respectively. Conclusions: (1) Fasting glycemia diagnostic for GDM measured in early pregnancy is associated with a significantly elevated risk for congenital malformations. (2) Our data suggest that women at elevated risks of GDM/diabetes in pregnancy (DiP) should have their fasting blood glucose assessed before becoming pregnant, and the optimization of glycemic control should be considered if the FBG exceeds 5.1 mmol/dL.

## 1. Introduction

Maternal hyperglycemia is a well-studied risk factor for fetal abnormalities. Clinical studies on diabetic pregnancies consistently report an increased proportion of fetal malformations in type 1 or type 2 diabetes-complicated pregnancies [1,2]. There is a common consensus—supported by data from animal models on the mechanism of teratogenic action of maternal hyperglycemia—that clinically relevant hyperglycemia increases the risk of embryonic and fetal maldevelopment [3,4]. Therefore, preconception counselling in the diabetic population focuses mainly on achieving blood glucose levels as close as possible to those seen in nonpregnant, normoglycemic women, provided that such an intensification of metabolic control does not put a woman at risk of hypoglycemic complications.

Unfortunately, elevated blood glucose is a continuous risk factor without a clear cut-off value discriminating low-risk individuals from those at high risk. Thus, despite the considerable improvement in metabolic control and treatment, the proportion of early pregnancy complications still remains elevated in women with pregestational diabetes who achieve a good level of metabolic control before their pregnancies [5,6]. The disease remains associated with an elevated risk of fetal malformations, even if novel therapies or glycemic surveillance methods are used [7,8].

However, as fasting glucose measurements at the first visit during pregnancy became a part of the standard antenatal care offered for the general population in many countries, we are now facing a new clinical problem of hyperglycemia detected in early pregnancy in women without a history of diabetes mellitus or prediabetes [9]. The question as to whether this biochemical finding is clinically relevant raises several controversies, as we still lack a consensus on normal blood glucose in early pregnancy [10,11]. The Hyperglycemia and Adverse Pregnancy Outcome (HAPO) study linked mid-pregnancy glucose levels in 75-g OGTT to the risk of certain maternal or neonatal complications and recommended diagnostic thresholds for gestational diabetes mellitus, later adopted by an international consensus [12,13]. However, these cut-off points were identified at the gestational ages of 24–28 weeks for the specific adverse outcomes typically resulting from hyperglycemia in the second half of pregnancy. There is the paucity of data validating the use of the International Association of the Diabetes and Pregnancy Study Groups (IADPSG) criteria in early pregnancy for predicting gestational diabetes mellitus (GDM) in late-pregnancy or reducing adverse neonatal outcomes [6,14,15]. Hormonal alterations that are typical of pregnancy change the glycemic response throughout gestation. Therefore, some researchers suggested that thresholds identified for the early third trimester of pregnancy are inappropriate to use in early pregnancy [16,17,18].

Standards of the Polish Society of Obstetrician and Gynaecologists (PSOG) adopted in 2014 recommend testing for GDM according to the IADPSG protocols and criteria not only in the third trimester for all pregnant women without pregestational (type 1 or type 2) diabetes mellitus but, also, in early pregnancy, for (1) normoglycemic women who are at elevated risk of GDM (positive for at least one from the following: body mass index (BMI) ≥ 30 kg/m^2^, history of GDM/macrosomia in previous pregnancies, history of polycystic ovary syndrome (PCOS), or insulin resistance) or (2) pregnant women presenting with fasting glycemia above 5.1 mmol/dL at the first trimester antenatal visit [19]. Moreover, previous PSOG recommendations, issued in 2011, also confirmed using fasting glycemia routinely measured in the general pregnant population in the first trimester of pregnancy as a screening test: women with a fasting glycemia > 5.5 mmol/dL should be offered a single-step procedure of a diagnostic 75-g OGTT performed in early pregnancy [20]. Therefore, we were able to design an observational study linking the blood glucose levels in early pregnancy to neonatal complications specifically resulting from maternal hyperglycemia in the periconceptional period.

We hypothesized that IADPSG criteria used in early pregnancy are suitable to identify women at elevated risk of congenital malformations, which are severe fetal complications caused by maternal hyperglycemia in early pregnancy and the periconceptual period.

## 2. Experimental Section

### 2.1. Inclusion and Exclusion Criteria

To test our hypothesis, we designed a retrospective observational trial. All pregnant women in singleton pregnancies referred to our tertiary level of care unit dealing with pregnancy and diabetes (a department within the University Obstetrical and Gynaecological Hospital at the Poznan University of Medical Sciences Poznan, Poland, which serves a population of ca. 4 million of citizen, with ca 7500 deliveries per year) between 2007 and 2017 for further treatment because of hyperglycemia detected before a gestational age of 20 weeks were considered eligible for the study. We reviewed maternal and neonatal records and used the following exclusion criteria: multiple pregnancies, lacking maternal/neonatal data, miscarriage, late intrauterine death, and did not give birth in our hospital. Finally, we identified 125 mother-newborn pairs.

### 2.2. Patients’ Enrolment and Data Collection

Patients enrolled into the study had 75-g OGTT done in the first trimester of pregnancy, because they were qualified as having a high risk for GDM/diabetes in pregnancy (DiP) or presented for their first antenatal visit with a fasting glycemia above 5.1 mmol/dL if diagnosed following the IADPSG criteria (adopted in Poland in 2014) or above 5.5 mmol/dL if diagnosed before 2014. The patients were subsequently referred to our unit if the diagnostic 75-g OGTT was found abnormal, i.e., at least one value was above the thresholds: 100-(180—measurement not mandatory)-140 mg/dL for women diagnosed until 2014 or 92-180-153 mg/dl for women diagnosed after 2014. During the whole study period, women with no history of pregestational diabetes mellitus who presented with a fasting glycemia above 7.0 mmol/dL or with a random glucose level above 11.1 mmol/dL were immediately referred to our department with a diagnosis of hyperglycemia in early pregnancy, without the 75-g OGTT.

For purposes of our study, we defined gestational diabetes mellitus (GDM) or diabetes in pregnancy (DiP) using IADPSG criteria in the whole cohort [13].

Pre-pregnancy bodyweight and glycemic values during 75-g OGTT were retrieved from patients’ pregnancy charts during their first admission to the department and transferred to the hospital documentation and, thus, were available for the researchers reviewing the records. The same applied to the gestational age, which was confirmed with the first ultrasound scan available in patients’ documentation and also transferred to the documentation upon admission for the baseline hospitalization. All participants had their scan for genetic risk assessment done between the 11th and 13th gestational weeks, and none of them required invasive diagnostic procedures due to an increased risk of trisomies. In the mid-trimester scan, four women were diagnosed with fetal malformations, none of which were lethal. None of the patients opted for pregnancy termination.

HbA1c and lipids were measured at the first admission to our unit (at the baseline) in the central laboratory of the academic hospital, holding certificates of quality management ISO 9000. To get some information on the average maternal daily glucose, which allows for gestational age as a modulator of the maternal glycemic profile, we calculated the pregnancy-specific estimated average glucose (PeAG) using the formula provided by Law et al. [21] from the daily glycemic profile collected during the baseline hospitalization. During this admission, pregnant women participated in the training, which addressed specific issues concerning general and diabetic dietary requirements in pregnancy, glucose self-monitoring, and lifestyle interventions. If glucose readings exceeded the targets, we added a basal-bolus insulin treatment, and these patients received further training on insulin therapy.

From the neonatal records, we obtained data on neonatal outcomes concerning congenital malformations confirmed postpartum. The postpartum assessment of each newborn was performed by a pediatrician—a neonatologist, including a postpartum cardiac echocardiographic scan.

The Bioethics Committee at the Poznan University of Medical Sciences confirmed that our research was not a medical experiment, therefore exempting our study from the need to obtain the committee’s approval (decision No 1321/18).

### 2.3. Statisticala Analysis

We used SPSS 14.0 for Windows (SPSS Inc. Chicago, IL, USA) and Medcalc 19.0 (Medcalc Software, Mariakerke, Belgium) to perform the statistical analysis. Variables were tested for normality with the D’Agostino-Pearson test—all variables, apart from maternal age, were not normally distributed. We used the chi-square test to compare the categorical variables. ROC (receiver operating characteristic) curve analysis was used to calculate the diagnostic power of the predictors for congenital malformations in the offspring. Descriptive variables are presented as mean ± standard deviation, unless stated otherwise. Two-sided *p <* 0.05 was considered statistically significant.

Logistic regression models with malformation status yes/no as a dependent variable were built to identify predictors of congenital malformations in the study group. We then used a forward selection to build models, including maternal characteristics known as risk factors for neonatal malformations, as confounders. Testing for the goodness of fit of a logistic regression model was performed with the Hosmer-Lemeshow test.

We used the chi-square test to calculate the likelihood ratios and 95% confidence intervals for particular glycemic thresholds used in the general or pregnant population and the risk of fetal malformations.

To verify, whether the small size of our cohort would allow for statistically sound observations, we performed a post-hoc analysis using Medcalc 19.0 (Medcalc Software, Mariakerke, Belgium) The analysis revealed that, with a proportion of malformations of 8.8% detected in our cohort, a minimum sample size of 75 would be needed to detect a difference in the proportions of fetal malformations compared to the general European population (2.5%, according to the European network of population-based registries for the epidemiological surveillance of congenital anomalies, EUROCAT), with an alpha of 0.05 and power of 0.8 [22]. We also performed a sample size estimation based on data regarding the prevalence of congenital malformations (3.33%) available for the whole cohort of N = 8055 neonates born or referred to the hospital in 2017. With an alpha of 0.5 and power of 0.8, a minimum sample size of 120 participants would be needed to detect a difference in the proportions.

We also confirmed that our study was powered to find a statistically significant difference in the outcome for the fasting glucose above 6.6 mmol/dL (post-hoc power: 96.2%) and 2-h glucose above 11.7 mmol/dL (post-hoc power: 94.2%). The post-hoc power for HbA1c above 5.7% was 73.3%.

## 3. Results

Characteristics of the study group are summarized in Table 1.

The participants were mostly referred for early GDM testing by their obstetricians because of pre-pregnancy obesity (39.6%), hyperglycemia, defined as fasting blood glucose above 5.1 mmol/dL in the cohort tested according to IADPSG/5.5 mmol/dL in the cohort tested before the IADPSG criteria came into use (76.9%), and either/or GDM in a past pregnancy (24.6% of women who had delivered at least once). In 18.6% of patients, 75-g OGTT was skipped, because fasting glycemia exceeded the threshold for overt diabetes in the general population, according to the WHO (7.0 mmol/dL). The cohort consisted of similar proportions of women diagnosed with GDM before IADPSG criteria were recommended (49.1%) and after that (50.9%). These two subgroups did not differ significantly regarding maternal BMI or maternal age.

Eleven out of 125 women delivered newborns with congenital malformations (8.8%). Three of them were minor, i.e., two cases of cryptorchism and one case of a syndactylia of the second and third finger. We confirmed cardiac malformations in six, genitourinary defects in two and skeletal defects in two out of these eleven cases. In one newborn, we diagnosed multiple defects of the skeletal and central nervous system and gastrointestinal tract. Women who gave birth to affected infants did not differ from those who delivered healthy newborns in terms of pregnancy-specific estimated average glucose, maternal age, pre-pregnancy body weight, pre-pregnancy BMI, parity, or the prevalence of chronic hypertension. The prevalence of congenital malformation in the subgroup treated with a diet (8.5%) was similar to the proportion found in women who required insulin treatment (9.3%, *p* = 0.852). Additionally, newborns with malformations did not differ from healthy infants in terms of gestational age at the delivery and birth weight. No statistically significant difference in the proportion of congenital malformations was found between participants diagnosed according to the earlier criteria (until 2014) and those diagnosed according of the IADPSG criteria (5.7% vs. 9.8%, *p* = 0.483)

In seven out of 114 healthy newborns (6.1%), a neonatal cardiac assessment done postpartum found an open foramen ovale. However, this feature was not classified as a malformation. In a separate analysis, we found no differences comparing maternal anthropometric, metabolic, or glycemic characteristics between women who gave birth to healthy newborns with vs. without this cardio-sonographic finding.

None of the available maternal anthropometrics, i.e., maternal age, pre-pregnancy body weight, or pre-pregnancy BMI, were associated with an increased risk of fetal malformations.

Maternal lipids in the first trimester of pregnancy were not associated with an elevated risk of fetal malformations, except low HDL (high-density lipoprotein) cholesterol levels: the relative risk for congenital malformations was significantly higher in women with HDL ≤ 45 mg/dL (likelihood ratio (LR): 3.2 95% CI: 1.1–9.8).

Comparing glycemic parameters between women who gave birth to affected vs. healthy newborns, we found a significant difference between the groups in blood glucose levels during 75-g OGTT and HbA1c measured at the first admission to the referral unit (for fasting glucose: 8.8 ± 3.5 vs. 5.6 ± 0.8 mmol/dL, *p* = 0.001, for 2-h glucose: 13.0 ± 6.6 vs. 8.9 ± 2.4, *p* = 0.034 mmol/dL, and for HbA1c at the baseline: 6.6 ± 1.9 vs. 5.7 ± 1.0, *p* = 0.023). In the multiple logistic regression models, we were able to confirm that fasting glycemia in 75-g OGTT and HbA1c at the baseline remained statistically significant predictors of birth defects in the cohort and when other maternal parameters, known as risk factors for congenital malformations in the general population (maternal age and BMI), were included in the models (Table 2).

Being aware of the specific nature of maternal glycemia as a continuous risk factor for neonatal complications, we obtained cut-off points with an optimal specificity and sensitivity for our cohort from ROC curves (Table 3). We then calculated the relative risk for congenital malformations for different values used as diagnostic thresholds in the general or pregnant population. To define the thresholds that could be clinically useful for a risk assessment in early pregnancy in our cohort, we used the approach described by the HAPO group, i.e., calculated the glycemic thresholds related to the 75% increase in the risk for the outcome [12]. We also estimated the highest glycemic values, for which the risk for congenital malformations remained close to 1.00, i.e., it was similar to the unaffected subgroup (Table 4).

## 4. Discussion

Our study provides data from a specific population. Our cohort is heterogeneous: it is comprised of women qualified as being at high risk of carbohydrate intolerance due to obesity/other risk factors for hyperglycemia present before pregnancy, or women without pre-pregnancy risk factors but with elevated fasting glucose diagnosed in early pregnancy. Regarding both indications, we can expect this population of pregnant women to increase mainly due to an increased prevalence of overweightness/obesity and advanced procreation age [23].

We confirmed maternal glycemia as a preventable risk factor for a severe fetal condition in the group of pregnant women without a diagnosis of hyperglycemia before pregnancy. From our data, we concluded that a fasting glycemia diagnostic for GDM according to the IADPSG criteria is associated with a significantly increased risk of congenital anomalies if used in pregnant women tested for gestational hyperglycemia in early pregnancy. Considering the ongoing discussion about whether these criteria are useful outside the gestational age at which they were used in the HAPO study, our research supports the use of IADPSG criteria also in early pregnancy to assess the risk of complications characteristic of maternal hyperglycemia in early pregnancy/periconception.

By adopting a more rigid threshold for normoglycemia for women attempting to conceive, we could likely further reduce the risk of severe neonatal complications in women without a history of pregestational diabetes mellitus but meeting the criteria of the high risk for the GDM population. From our data, we believe that reducing maternal glycemia would allow the management of fetal risk in our societies, characterized by women of childbearing age becoming older and heavier, which means an increasing prevalence of fetal anomalies in clinically normoglycemic individuals [24,25,26]. Adopting more stringent criteria for fasting glycemia would improve the neonatal prognosis in an epidemiological milieu of increased maternal age, which is an unmodifiable risk factor for adverse perinatal outcomes, and excessive maternal body weight, which is extremely difficult to treat in a short-term perspective necessary to adopt in a woman of a more advanced procreative age. Moreover, aiming at stricter fasting glycemia to protect from congenital malformation would also reflect a natural decrease in maternal glycemia typical of normal early pregnancy [10]. Moreover, a recent meta-analysis from Parnell et al. confirmed that the elevated risk for congenital malformations was seen only in women with GDM and obesity [27]. The authors emphasized that glycemic derangement typical of pre-pregnancy obesity, which remains undiagnosed until pregnancy, might be an actual driving factor behind this association.

In our cohort, we also noted a birth weight distribution similar to that seen in the normal population. This striking observation supports the notion that improved glycemic control resulting from the early treatment of GDM is likely to mitigate a late-pregnancy metabolic risk arising from mild maternal hyperglycemia in the second half of pregnancy, but the teratogenic impact of hyperglycemia evidently occurs before a diagnosis is made and any treatment can be commenced. However, the normal birth weight in our cohort might raise some concerns, suggesting that what we actually see is also a suboptimal growth (a number of LGA (large for gestational age)/macrosomia cases smaller than expected for such a dysmetabolic cohort) reflecting a subclinical placental dysfunction in women with severe insulin resistance (metabolic syndrome) or asymptomatic dysglycemia undiagnosed until pregnancy. Moreover, over 10% of our cohort suffered from arterial hypertension, which is a well-known disease affecting placental function. From our data we concluded that women at an elevated risk of gestational hyperglycemia should be identified in the general population and offered preconceptional counseling, similar to that offered to women with pregestational diabetes mellitus. Our results suggest that women with a high risk for GDM/DiP could be encouraged to start lifestyle interventions that aim at lowering fasting blood glycemia. More large-scale research is necessary to define the target, but our calculations indicate an optimal value even below 4.4 mmol/dL. We believe that a threshold of 5.1 mmol/dL can be safely used before more refined data from adequately powered studies are available. Discussing fetomaternal safety given future procreation plans could also prompt testing for all components of the metabolic syndrome and initiate a medical treatment of insulin resistance, if present.

Our study has some limitations. Due to the retrospective nature of our research, we had no data to check for hyperinsulinemia or insulin resistance, which seems to be another risk factor for fetal malformations [28,29]. Data from other studies suggest that the prevalence of this condition is currently increasing among young women [30,31]. This metabolic trait could be a driving force behind the coexistence of high-normal glucose levels and congenital malformations, which we noted in our cohort. This hypothesis is supported by our observation of a low HDL level, which is also a marker of metabolic syndrome, as a predictor of congenital malformations.

Another limitation comes from both the retrospective nature of our study and a specific setting in which our research was performed. We sampled the high-risk population of patients referred to the tertiary level of care unit. A proportion of malformations recorded for our hospital in 2017 was 3.3%—which is significantly larger than for a general population and reflects the specific nature of a large, referral academic center taking care for high-risk pregnancies. To appropriately assess an actual proportion of excess congenital malformations, prospective studies would be necessary with a specifically designed high-risk control group, i.e., pregnant women at the high risk for hyperglycemia in pregnancy but who were tested negative for GDM/DiP in early pregnancy.

As we mentioned above, our cohort is heterogeneous; it is comprised of women screened for GDM/DiP in early pregnancy because of their high-risk profiles known before pregnancy but, also, of women who did not present with any risk factors for GDM/DiP but were referred for further tests because of the elevated fasting glycemia found in early pregnancy. Such a biochemical finding can be noted in a healthy pregnancy as a result of prolonged overnight fasting. However, our study confirmed that the fasting glycemia was the only predictor for congenital malformations; maternal body weight/BMI itself did not increase the risk of birth defects in this very specific cohort. This suggests that a “high-normal” fasting blood glucose has a predictive value both in women with a pre-pregnancy high-risk profile and a low-risk profile concerning the GDM/DiP risk.

Elevated fasting glycemia in a pregnant woman not otherwise qualifying for early GDM/DiP testing can indicate an early stage of an autoimmune process against pancreatic beta cells or MODY (Maturity-onset diabetes of the young). Data on the prevalence of anty-islet antibodies among women with gestational diabetes are inconsistent: numbers reported for different populations vary from 6% to as high as 44% [32,33,34]. Our study provides another reason for such tests to be carried out in women with hyperglycemia detected in early pregnancy, especially if they meet the criteria of DIP. Even if intensive treatment and surveillance throughout pregnancy mitigate a short-term fetomaternal risk, such information is critical for planning future care and discussing prognosis with a patient.

We confirm that even “normal” fasting glycemia might be a reason for a “background rate” of congenital malformations. Due to a poor prognosis and the seriousness of this entity, every attempt should be made to avert as many cases of birth defects as possible. Although our center covers a large population of ca 4 million people, multicenter, large-scale studies are necessary to investigate the glycemic thresholds for particular BMI and ages, because a combination of these two risks can have an impact on the actual glycemic level necessary to optimize the risk. Additionally, from such studies, one could obtain information about a high-normal glycemia as an additional risk for early pregnancy loss.

The question as to whether women at low risk for GDM/DiP and a high-normal fasting glycemia should also be offered any pre-pregnancy intervention for further reducing their blood glucose, remains open. Although we can expect health benefits in women at high-risk for gestational hyperglycemia because their metabolic traits also increase the risk for premature cardiovascular morbidity, no such data is available for women of low cardiometabolic risk. However, these women could benefit from early testing towards anti-islet autoimmunity.

Data from our cohort suggest that fasting glycemia should be measured routinely in women who wish to get pregnant, mainly in populations with an increased prevalence of overweightness/obesity. Interventions to optimize fasting glycemia could then be initiated for women with fasting blood glucose above 5.1 mg/dL to reduce the risk of congenital malformations. Further, large-scale studies are warranted to investigate an optimal age- and BMI-specific fasting glycemia in view of the risk of congenital malformations and any specific pre-pregnancy interventions.

## Figures and Tables

**Table 1:** Characteristics of the study group (N = 125 mother-infant pairs); non-normally distributed variables are presented as medians (interquartile range) or %. BMI: body mass index, IADPSG: International Association of the Diabetes and Pregnancy Study Groups, and DiP: diabetes in pregnancy; IDF: International Diabetes Federation.

Maternal age (years)	31.5 ± 5.2
Pre-pregnancy body weight (kg)	81.1 ± 19.6
Pre-pregnancy BMI (kg/m^2^)	29.1 ± 6.5
% of women with pre-pregnancy BMI ≥ 30 kg/m^2^	42.0%
Gestational age at diagnosis (weeks)	12.0 ± 4.0
75-g OGTT fasting glycemia (mg/dL)/(mmol/dL)	101.0 (94.0; 112.5)/ 5.6 (3.5; 6.2)
75-g OGTT 120′ glycemia (mg/dL)/(mmol/dL) available for 91 participants	160.0 (135.0; 188.0)/ 8.9 (7.5; 10.4)
75-g OGTT 60′ glycemia (mg/dL)/(mmol/dL) available for 42 participants	168.0 (141.0; 192.0)/ 9.3 (7.8; 10.7)
% of women with DiP according to the IADPSG criteria [10]	21.5%
HbA1c (%) at baseline	5.5 (5.1; 6.0)
HDL cholesterol (mg/dL) at baseline	64.0 (54.7; 79.0)
% of women on diet and insulin	60.0%
% of women with Hba1c ≥ 6.5% at admission time	14.4%
% of women with chronic hypertension	11.2%
% of women meeting criteria of metabolic syndrome according to the IDF criteria at the baseline	17.6%
% of newborns with congenital malformations in the cohort	8.8%
% of healthy newborns with persistent foramen ovale	6.1%
Gestational age at delivery	38 (38; 39)
% of premature deliveries (delivery before 37th gestational age completed)	9.5%
Birth weight (g)	3420 (3080; 3756)
Birth weight > 4000 g (%)	12.8%

**Table 2:** Predictors for congenital malformations—data from multivariate logistic regression models.

Parameter	expB	95% CI for expB	*p*	R2 (Nagelkerke)
75-g OGTT fasting (mg/dL)	1.06	1.02–1.10	0.007	0.412
75-g OGTT 2 h (mg/dL)	1.02	0.99–1.04	0.09	0.197
HbA1c at baseline	2.05	1.24–3.38	0.005	0.191

After adjustment for maternal age, the pre-pregnancy body weight, pre-pregnancy BMI, and parity and gestational age at diagnosis.

**Table 3:** ROC curves for predictors of congenital malformations in the cohort. AUC: area under the curve and FBG: fasting blood glycemia.

Parameter	AUC	*p*	Sensitivity	Specificity
75-g OGTT FBG > 6.6 mmol/dL	0.82	0.002	77.8	90.1
HbA1c > 5.7%	0.72	0.035	80.0	71.2
75-g OGTT 2-h > 11.7 mmol/dL	0.73	0.093	62.5	94.0

**Table 4:** The risk for congenital malformations for different diagnostic thresholds; the risk is computed from glycemic data collected for our cohort; the thresholds are linked to the likelihood ratio (LR) (95% CI).

	75-g OGTT Fasting	75-g OGTT 2-h	HbA1c
Cut-off values from ROC curves constructed for our cohort;	6.6 mmol/dL	11.7 mmol. dL	5.7%
	^(LR)^ 7.9 (3.9–16.0)	10.4 (3.8–28.3)	2.8 (1.8–4.2)
The upper limit for normal fasting glycemia in the general population	5.5 mmol/dL	--	--
	^(LR)^ 1.5 (1.0–2.3)		
Diagnostic thresholds for GDM according to 2013 the WHO/IADPSG criteria	5.1 mmol/dL	8.5 mmol/dL	--
	^(LR)^ 1.3 (1.1–1.4)	1.4 (0.9–2.2)	
Diagnostic thresholds for DiP according to the WHO/IADPSG criteria	7.0 mmol/dL	11.1 mmol/dL	6.5% *
	^(LR)^ 12.1 (4.6–32.0)	7.4 (3.0–18.0)	*4.0* *(1.6–10.4)*
The HAPO Study approach—glycemic thresholds nearest to the OR of 1.75 in our cohort (approximated)	5.6 mmol/dL	9.2 mmol/dL	5.5% **
	^(LR)^ 1.73 (1.1–2.6)	1.73 (1.1–2.8)	*1.78* *(1.2–2.6)*
Glycemic thresholds nearest to the OR of 1.0 in our cohort (approximated)	4.2 mmol/ dL	6.1 mmol/dL	4.9%
	^(LR)^ 1.05 (1.00–1.09)	0.99 (0.76–1.31)	1.03 (0.83–1.28)

* According to the ADA (American Diabetes Association), HbA1c ≥ 6.5% can be used as a diagnostic for overt diabetes in the general population; 75-g OGTT 1-h glycemia was not used for calculations, because this value was available only in a small number of patients. ** The Hyperglycemia and Adverse Pregnancy Outcome (HAPO) study did not provide odds ratios (ORs) for the HbA1c levels. GDM: gestational diabetes mellitus.

## Abbreviations

ADA—American Diabetes Association, AUC—area under curve, DiP—diabetes in pregnancy, HbA1c—haemoglobin A1c (glycated haemoglobin), GDM—gestational diabetes mellitus, FBG—fasting blood glycemia, HAPO Study—Hyperglycemia and Adverse Pregnancy Outcome Study, HDL—high-density lipoprotein, IADPSG—International Association of the Diabetes and Pregnancy Study Groups, IDF—International Diabetes Federation, LGA—large for gestational age, LR—likelihood ratio, MODY—Maturity-onset diabetes of the young, OGTT—oral glucose tolerance test; PCOS—polycystic ovary syndrome, and PeAG—pregnancy-specific estimated average glucose, ROC curve—receiver operating characteristic curve.
